# Long-acting intranasal insulin for the treatment of delirium—a randomised clinical trial

**DOI:** 10.1093/ageing/afaf276

**Published:** 2025-10-14

**Authors:** Anita Nitchingham, Jacqueline C T Close, Lara Ann Harvey, Morag E Taylor, Peter Humburg, Bernard Tuch, Meera Agar, Gideon A Caplan

**Affiliations:** Randwick Clinical Campus, University of New South Wales, Randwick, NSW, Australia; Department of Geriatric Medicine, Prince of Wales Hospital and Community Health Services, Randwick, NSW, Australia; Falls, Balance and Injury Research Centre, Neuroscience Research Australia, Randwick, NSW, Australia; Randwick Clinical Campus, University of New South Wales, Randwick, NSW, Australia; Department of Geriatric Medicine, Prince of Wales Hospital and Community Health Services, Randwick, NSW, Australia; Falls, Balance and Injury Research Centre, Neuroscience Research Australia, Randwick, NSW, Australia; Falls, Balance and Injury Research Centre, Neuroscience Research Australia, Randwick, NSW, Australia; School of Public Health and Community Medicine, University of New South Wales, Sydney, NSW, Australia; Falls, Balance and Injury Research Centre, Neuroscience Research Australia, Randwick, NSW, Australia; School of Health Sciences, University of New South Wales, Sydney, NSW, Australia; Ageing Futures Institute, University of New South Wales, Sydney, NSW, Australia; Mark Wainwright Analytical Centre, University of New South Wales, Sydney, NSW, Australia; Department of Diabetes, Central Clinical School, Monash University, Melbourne, VIC, Australia; IMPACCT, Faculty of Health, University of Technology Sydney, Sydney, NSW, Australia; Randwick Clinical Campus, University of New South Wales, Randwick, NSW, Australia; Department of Geriatric Medicine, Prince of Wales Hospital and Community Health Services, Randwick, NSW, Australia

**Keywords:** delirium, treatment, intranasal insulin, pharmacological, geriatrics, older people

## Abstract

**Background:**

Delirium affects up to 25% of hospitalised older patients; however, there are no effective pharmacological treatments. Accumulating evidence of brain insulin resistance and altered cerebral glucose metabolism during delirium present a promising therapeutic target.

**Objective:**

To assess the safety and efficacy of intranasal insulin in the treatment of delirium.

**Design:**

Single-centre, randomised, double-blind, placebo-controlled trial.

**Setting:**

Two geriatric medicine wards in a tertiary hospital.

**Participants:**

100 patients aged over 64 years presenting to hospital and admitted under geriatric medicine with delirium.

**Interventions:**

Participants were randomised in a 1:1 ratio to receive 20 IU of long-acting insulin or placebo intranasally twice daily until delirium resolution, hospital discharge or intervention futility defined by prespecified criteria.

**Main outcome:**

The primary outcome was delirium duration, assessed daily using the Confusion Assessment Method. Secondary outcomes included acute length of stay (LOS), delirium severity, antipsychotic use, hospital complications and mortality.

**Results:**

The intention-to-treat analysis included 97 participants [intranasal insulin *n* = 48, control *n* = 49; mean (SD) age, 87.6 (7.0) years; 63% female]. Baseline characteristics were similar between groups. Median delirium duration [days (IQR)] was 4.8 [2.9, 9.2] for intranasal insulin and 6.8 [4.0, 9.8] for the control (HR 0.7, 95% CI 0.43–1.15; *P* = .16). Median acute LOS (days) was 7.9 [4.6, 14.5] for intranasal insulin and 12.9 [6.9, 16.8] for the control (HR 0.56, 0.35–0.89; *P* = .014). No significant differences were observed in other secondary outcomes. Intranasal insulin demonstrated favourable tolerability. Overall, 86% of the participants were compliant with the intervention (≥80% of doses). Prespecified subgroup analysis revealed an age-related response, with participants aged ≤88 years showing shorter delirium duration with intranasal insulin [*n* = 46; intranasal insulin: median 3.9 (IQR 2.9, 6.9) days vs control: 7.0 (4.7, 9.7); HR 0.34, 0.16–0.74; *P* = .006], whereas no difference was observed in participants aged >88 years [*n* = 51; intranasal insulin 5.4 (2.9, 11.1) vs control 4.9 (2.6, 12.9); HR 0.87, 0.39–1.94; *P* = .73].

**Conclusion and relevance:**

This is the first study of intranasal insulin for delirium treatment. The reduced LOS combined with the observed age-related effects warrants further investigation into the clinical potential of intranasal insulin in managing delirium in older patients.

**Trial registration:**

ACTRN 12618000318280.

## Key Points

- Delirium affects up to 25% of older hospitalised patients; however, there are no licensed treatments.
- Brain insulin resistance and altered glucose metabolism may be therapeutic targets.
- Intranasal insulin did not reduce delirium duration but was associated with shorter hospital stay.
- An age-related response was observed, warranting further investigation.

## Background

Delirium, a prevalent and distressing acute neurocognitive disorder, remains without licensed pharmacological treatment despite its impact on patients, caregivers and healthcare systems. Affecting up to 31% of older adults presenting to hospital, delirium is associated with hospital-acquired complications, including falls, longer hospital stays, functional decline and death [1–4]. While delirium may be transient with rapid resolution within hours to days, in other cases, delirium persists for weeks to months, ultimately leading to an irreversible decline in cognition and function [5]. For many older people, an episode of delirium is a critical event leading to increased functional dependence, institutionalisation and diminished quality of life [6].

Despite the success of multimodal prevention strategies [7], these same interventions, when implemented following the onset of delirium, do not appear to alter its trajectory [8]. There are no medications for the prevention or treatment of delirium on general hospital wards. Antipsychotics are sometimes used to manage distressing symptoms; however, studies have consistently shown that they do not improve outcomes and may cause harm, underscoring the need for alternative therapies [9, 10].

Growing evidence shows altered cerebral glucose metabolism and brain insulin resistance during delirium in older people, presenting a tangible therapeutic target [11–16]. In the brain, insulin facilitates glucose transport via glucose transporter type 4 and may also activate glucose transporter type 3, thereby supporting neuronal energy supply [17, 18]. Beyond this, insulin has neuromodulatory effects on neuroinflammation, functional connectivity and the hypothalamic–pituitary–adrenal axis—all pathways implicated in delirium [19–22].

Intranasal insulin delivers insulin directly into the central nervous system, bypassing the blood–brain barrier without causing clinically significant systemic glucose effects at moderate doses [23–25]. It is well tolerated, with side effects primarily limited to nasal irritation—an important consideration for frail, medically complex older adults [26]. Single-centre studies suggest it may be effective in preventing postoperative delirium [27–32]. However, no studies to date have investigated its use in treating established delirium.

In this novel study, we investigated the safety and systematic efficacy of long-acting intranasal insulin for the treatment of delirium. We hypothesised that intranasal insulin could reduce the duration of delirium compared to placebo in older people presenting to hospital with delirium being treated on aged care wards.

## Methods

### Trial design and oversight

This was a single-site, double-blind, randomised, placebo-controlled trial conducted at a 450-bed tertiary hospital in Sydney, Australia. Approvals were obtained from both the New South Wales Civil and Administrative Tribunal (2017/00204946) and the South Eastern Sydney Local Health District Human Research Ethics Committee (HREC) (16/320). The study was registered prospectively on the Australian New Zealand Clinical Trials Registry (ACTRN 12618000318280). The trial protocol is published [33]. A data and safety monitoring board (DSMB), comprising two geriatricians and an endocrinologist, oversaw the study and met after each 20 participants for the first 40 participants, and then at intervals at their discretion. This trial is reported in accordance with the Consolidated Standards of Reporting Trials (CONSORT) checklist.

### Participants

Patients were eligible if they met the following criteria: (i) they had prevalent delirium on admission to hospital, diagnosed by a geriatrician or advanced trainee in geriatric medicine using the *Diagnostic and Statistical Manual of Mental Disorders, Fifth Edition* (*DSM-5*) criteria [34]; (ii) they were receiving care on geriatric medicine wards and under the care of a geriatric medicine team; (iii) their age was >64 years; (iv) they had a consenting ‘Person Responsible’ (substitute decision-maker according to the New South Wales Guardianship Act of 1987); and (v) they enrolled in the trial within 48 hours of admission to hospital. Initially, enrolment was required within 24 hours; however, the protocol was extended to 48 hours during the COVID-19 pandemic due to recruitment delays related to COVID-19 testing wait times.

The exclusion criteria were as follows: (i) haemodynamically unstable (based on treating physician’s judgement guided by activation of a ‘red zone response’ on the New South Wales Health Standard Adult General Observation Chart), (ii) life expectancy < 7 days as judged by the admitting geriatrician, (iii) allergy to insulin detemir, (iv) structural abnormality precluding use of the nasal drug delivery device or (v) proven or suspected COVID-19. Patients unable to participate in cognitive assessments due to limited English proficiency were also excluded. Patients with mild cognitive impairment and dementia were not excluded.

When eligible participants were identified, research staff contacted their Person Responsible to provide study information and obtain consent. To avoid delays, verbal consent was granted via phone, followed by written consent as soon as possible. Consent to remain in the trial was obtained from the participant if their capacity returned.

### Randomisation and blinding

An independent clinical trials pharmacist created a computer-generated permutated block randomisation schedule (block size of 4 and 25 blocks). This was provided to the clinical trials pharmacy staff responsible for production and dispensing. Vials of insulin or placebo were labelled with a sequentially allocated randomisation number, which became the participant’s study identification number. Vials were kept in the ward fridge, available for use when a patient was recruited.

Participants, research staff and ward staff were blinded to treatment allocation. The placebo and insulin were a clear fluid, identical in appearance.

### Intervention

Participants received 20 international units (IU) of long-acting insulin (insulin detemir, Levemir® Novo Nordisk) or placebo (normal saline) intranasally twice daily at 8 a.m. and 8 p.m. through a ViaNase™ delivery device (Kurve Therapeutics, Bothell, WA, USA). The device delivered 20 IU of insulin over 40 seconds; participants were prescribed 20 seconds of insulin or placebo into both nostrils twice daily. Research geriatricians prescribed the intervention onto the electronic medical chart and trained ward nurses administered it and documented adherence, including reasons for missed or partial doses.

The intervention ceased upon delirium resolution, defined as two consecutive days with a negative confusion assessment method (CAM-Long) [35]. Given the high prevalence of intercurrent dementia, collateral history and caregiver observations supported the assessment of whether participants had returned to their cognitive baseline. Cessation also occurred if (i) the participant was discharged; (ii) the participant or treating clinician requested discontinuation; (iii) there were unacceptable side effects [defined by the National Cancer Institute Common Terminology Criteria for Adverse Events (CTCAE) version 4.0]; (iv) the investigator deemed the participant not well enough to continue; (v) there were adverse events related to the study medication that were unacceptable to the participant/carer or clinician; or (vi) the treatment was ineffective, defined as no improvement in delirium severity measured by the delirium index (DI) over 7 days [36].

### Data collection

Demographic data were collected by research staff at trial enrolment and extracted from medical records and collateral history. The data included age, sex, education, residence, medical history and medications on admission. Frailty was assessed using the Clinical Frailty Scale (CFS) and chronic disease burden by the Charlson comorbidity index (CCI) [37, 38]. The severity of acute illness was measured using Acute Physiology and Chronic Health Evaluation (APACHE) III [39]. Functional status was evaluated using the Barthel Index and Instrumental Activities of Daily Living (IADL) index [40, 41]. Baseline cognition was assessed using the Informant Questionnaire on Cognitive Decline in the Elderly (IQCODE) [42].

### Primary outcomes

The primary outcome was duration of delirium in days, measured from the time of hospital admission until midday on the first of two consecutive CAM-negative days. In cases of fluctuating CAM results (e.g. CAM positive → CAM negative → CAM positive), delirium was considered ongoing, and resolution was not assigned until sustained improvement was observed. This approach minimised misclassification of transient recovery and accounted for the fluctuating nature of delirium. A structured delirium assessment was conducted daily, between 12 and 3 p.m., by a trained researcher until delirium resolution. The majority of bedside assessments were conducted by two researchers. Where there was uncertainty regarding delirium resolution, participants were reviewed by the principal investigator (G.C.). Participants discharged with delirium were followed up daily, in person at their home or subacute health facility, for up to 1 week to assess for delirium resolution.

### Secondary outcomes

Secondary outcomes included the effect of intranasal insulin on acute length of stay, delirium severity over time, hospital complications defined by the Australian Commission on Safety and Quality in Health Care list, new antipsychotic use, new admission to a nursing home and inpatient and 30-day mortality. Functional and cognitive data from 6-month follow-up will be evaluated in a separate article. Patients were defined as compliant if ≥80% of doses were successfully administered [43].

### Adverse events

Researchers defined and graded adverse events daily using the CTCAE version 4, through participant interview and review of the medical record. Serious adverse events were discussed with the principal investigator immediately and reported to the HREC and DSMB within 24 hours.

### Sample size calculation

The study was powered based on data demonstrating a mean delirium duration of 8 days on geriatrics wards [44, 45]. Assuming a 30% attrition rate, 100 participants were required to detect a 2-day reduction in delirium duration with 80% power and 5% significance. As longer duration of delirium predicts poorer outcomes [46–48], in the absence of robust evidence, a 2-day reduction was considered clinically significant, with potential benefits for patient outcomes and healthcare utilisation [49].

### Statistical analysis

A statistical analysis plan was published prior to unblinding [50]. Data were analysed with SPSS Statistics 26 and SAS Enterprise Guide 8.2. Categorical data are presented as frequencies and proportions; continuous data as mean ± SD or median [interquartile range (IQR)], as appropriate. Outliers for continuous dependent variables were capped at 3 SD above the mean (duration of delirium, control *n* = 2; acute length of stay, intranasal insulin *n* = 1, control *n* = 1) [51]. The analyses followed an intention-to-treat (ITT) approach, with participants allocated to their randomised group, regardless of protocol compliance.

The primary outcome, duration of delirium (days), was analysed with a Mann–Whitney *U* test and Cox proportional hazard model, adjusting for age, sex, dementia (IQCODE > 3.44), nursing home status, APACHE, CFS and CCI, with age adjustments removed from the APACHE and CCI. Subgroup analysis was performed by age, sex, history of dementia (IQCODE > 3.44) and frailty (CFS ≥ 5) using the adjusted Cox model. Given the cohort’s advanced age, we dichotomised age using the cohort median (median = 88.4 years and therefore age ≤ 88 vs >88 years), a data-driven and pragmatic approach to preserve power when analysing continuous variables [52, 53]. A *post hoc* subgroup analysis using the same model with an age cut-off of 85 years [54] is presented in Appendix A (see online supplementary material*)*.

Sensitivity analysis included an adjusted subdistribution hazards model with death as a competing risk. Per-protocol analysis was conducted using the adjusted Cox proportional hazards model. Prespecified exploratory analysis assessed response to intranasal insulin by delirium precipitant using an adjusted subdistribution hazards model accounting for death as a competing risk.

Unadjusted analysis of the primary outcome was blinded, and the subsequent statistical analysis was unblinded.

#### Secondary outcomes

Multivariable models examining delirium severity, length of stay, hospital complications, new admission to skilled nursing facility and mortality were adjusted for age, sex, frailty, cognition, place of residence, APACHE and comorbidity. Delirium severity (up to Day 7) was analysed using a generalised linear mixed model. The last DI score was carried forward for resolved cases and the maximum score was assigned for deceased participants. Length of hospital stay was assessed using a gamma regression with log link. Total hospital complications were analysed with negative binomial regression, and rates of complications, skilled nursing admission and mortality with modified Poisson regression [55].

The proportion of participants receiving new antipsychotics was assessed using a chi-squared test and differences in antipsychotic dose equivalence with a Mann–Whitney *U* test.

All the tests were two-sided, with significance set at *P* < .05.

## Results

### Recruitment

**Figure 1:**
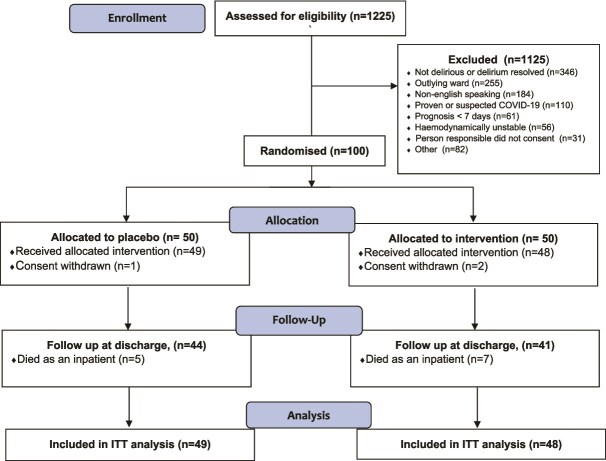
CONSORT 2010 flow diagram.

Between March 2018 and March 2023, 1225 patients were screened for eligibility. One break in recruitment occurred from May 2019 to June 2020 due to staff leave and COVID-19. Recruitment and ineligibility are summarised in Figure 1. The most common reasons for exclusion were that the patient was not delirious or the delirium had resolved (31%), or the patient was being cared for on an outlying ward or had proven or suspected COVID-19 (30%). One hundred participants were randomised. Three participants withdrew consent after randomisation, leaving 97 participants (insulin *n* = 48, control *n* = 49) in the ITT analysis.

The participants’ characteristics are reported in Table 1. The groups were well matched in terms of age, frailty and comorbidity; however, there were more females in the control group, *P* < .01. Thirty percent of the cohort had a documented history of dementia; however, based on the IQCODE, a higher proportion likely had undiagnosed dementia. The most common motoric delirium subtype was hypoactive and the most common precipitant was infection.

**Table 1:** Patient characteristics, *n* (%) or mean ± SD.

Characteristic	Total	Intranasal insulin	Control
Participants, no.	97	48	49
Age, years	87.6 ± 7.0	88.3 ± 7.5	86.8 ± 6.5
Sex, female	61 (63)	23 (47.9)	38 (77.6)
Education			
Up to primary	14 (14)	9 (19)	5 (10)
Up to secondary	56 (58)	29 (60)	27 (55)
Tertiary	27 (28)	10 (21)	17 (35)
Accommodation			
Community dwelling	79 (81)	38 (79)	41 (84)
Long-term care facility	18 (19)	10 (21)	8 (16)
CFS	5.6 ± 1.0	5.6 ± 1.1	5.5 ± 0.9
CCI	6.2 ± 1.9	6.4 ± 1.9	6.1 ± 1.8
APACHE III	45.4 ± 10.7	47.0 ± 11.7	43.8 ± 9.4
iADL index	4.2 ± 3.6	3.9 ± 3.8	4.4 ± 3.4
Barthels Index	14.8 ± 5.3	13.9 ± 5.5	15.6 ± 4.9
No. medications on admission	8.8 ± 4.6	9.2 ± 4.8	8.5 ± 4.4
Medical history			
IHD	22 (23)	12 (25)	10 (20)
Diabetes	22 (23)	11 (23)	11 (23)
Stroke	9 (9)	4 (8)	5 (10)
History of dementia	30 (31)	12 (25)	18 (37)
IQCODE ≤ 3.44	18 (19)	9 (19)	9 (18)
Delirium motoric subtype			
Hyperactive	17 (18)	9 (19)	8 (16)
Hypoactive	41 (42)	21 (44)	20 (41)
Mixed	39 (40)	18 (38)	21 (43)
Primary delirium precipitant^a^			
Infection	49 (51)	23 (48)	26 (53)
Electrolyte disturbance^b^	12 (12)	5 (10)	7 (14)
Medications	6 (6)	3 (6)	3 (6)
Neurological^c^	8 (8)	5 (10)	3 (6)
Other^d^	16 (17)	9 (19)	7 (14)
Unknown	6 (6)	3 (6)	3 (6)

a Most had multiple causes of delirium.

b Includes renal failure.

c Includes ischaemic and haemorrhagic stroke, Guillain-Barre, encephalitis.

d Includes urinary retention, constipation, thyroid disorders, fractures, pain.

Abbreviations: APACHE III, Acute Physiology and Chronic Health Evaluation III; CCI, Charlson Comorbidity Index; CSF, Clinical Frailty Scale; COPD, chronic obstructive pulmonary disease; iADL, Instrumental Activities of Daily Living Index; IHD, ischaemic heart disease; IQCODE, Informant Questionnaire on Cognitive Decline in the Elderly.

### Primary outcomes

**Figure 2:**
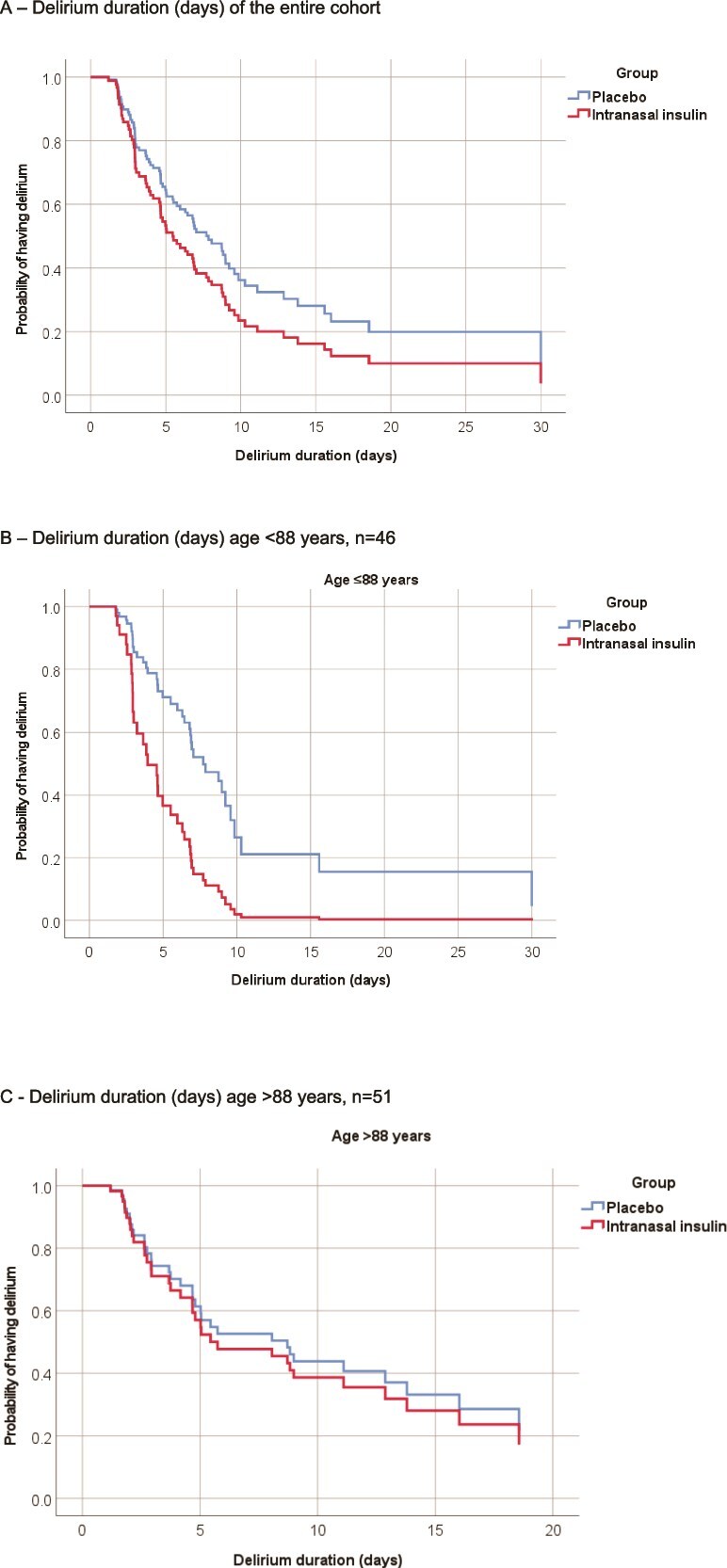
Analysis of delirium duration. (A) Delirium duration (days) of the entire cohort. (B) Delirium duration (days) age < 88 years, *n* = 46. (C) Delirium duration (days) age > 88 years, *n* = 51. Time to resolution of delirium modelled using multivariable Cox regression. Intranasal insulin vs placebo. (A) Entire cohort: *P* = .16. (B) Study participants aged ≤88 years: *P* = .006. (C) Study participants aged >88 years: *P* = .73.

In the primary ITT analyses, the median (IQR) days of delirium were 4.82 (2.93, 9.21) in the insulin arm and 6.79 (3.97, 9.76) in the control arm [Mann–Whitney *U*: *P* = .27, unadjusted hazard ratio (HR) 0.9; CI 0.57–1.43; *P* = .66, adjusted HR 0.7; CI 0.43–1.15; *P* = .16] (Figure 2).

On prespecified subgroup analysis, an age-related response was observed. Participants aged ≤88 years demonstrated a significant reduction in delirium duration [insulin: 3.88 (2.87, 6.92) days vs control: 7.03 (4.67, 9.66) days, HR 0.34, CI 0.16–0.74; *P* = 0.006], while no difference was observed in participants aged >88 years [insulin: 5.44 (2.94, 11.11) days vs control: 4.91 (2.64, 12.87) days, HR 0.87, CI 0.39–1.94, *P* = .73] (see Figure 2). Subgroup analysis using an age cut-off of ≤85 years is presented in Appendix A (see online supplementary material).

Subgroup analysis by sex revealed no significant differences. Subgroup analysis by frailty status and IQCODE was not possible as there were too few people in the nonfrail (*n* = 12) and cognitively intact groups (IQCODE ≤ 3.44, *n* = 18).

### Secondary outcomes

Secondary outcome data are presented in Table 2. There was no difference in delirium severity between groups. Participants in the intranasal insulin group had a shorter acute length of stay, median days (IQR) 7.93 (4.62, 14.48) for insulin and 12.91 (6.86, 16.79) for the control (HR 0.56, CI 0.35–0.89, *P* = .014). Thirty percent of the cohort experienced at least one hospital-acquired complication; there was no difference between groups. Antipsychotic use was similar between groups.

**Table 2:** Secondary outcome data, *n* (%), mean ± SD or median [IQR]

	No. (%)			
Characteristic	Total, *n* = 97	Intervention, *n* = 48	Control, *n* = 49	Statistic
Improvement in baseline DI days 1–7	5.09 ± 4.5	5.29 ± 4.1	4.9 ± 4.0	*B* ^a^ = −0.87; 95% CI: −2.79 to 1.06; *P* = .38
Acute length of stay, days	10.0 [5.1, 16.0]	7.9 [4.6, 14.5]	12.91 [6.9, 16.78]	HR^a^ 0.56; 95% CI, 0.35–0.89; *P* = .014
Hospital-acquired complications (HAC)				
Patient experiencing ≥1 HAC	30 (30)	16 (33)	14 (29)	RR^a^ 0.87; 95% CI, 0.46–1.65; *P* = .67
Total number of HACs	50	21	29	IRR^a^ 0.56; 95% CI, 0.25–1.25; *P* = .16
Pressure injury	3	1	2	
Fall	18	7	11	
Infection	6	3	3	
Respiratory complications	8	4	4	
Hypoglycaemia	8	4	4	
Other	7	2	5	
Antipsychotic use				
New antipsychotic prescription	15 (16)	8 (17)	7 (14)	*P* = .75
Dose-equivalent risperidone per day, mg	0.15 [0.11–0.54]	0.18 [0.14–0.51]	0.15 [0.08–0.74]	*P* = .62
In-hospital mortality	12 (12)	7 (15)	5 (10)	RR^a^ 0.66; 95% CI, 0.17–2.48; *P* = .54
30-day mortality	14 (14)	8 (17)	6 (12)	RR^a^ 0.72; 95% CI, 0.26–2.10; *P* = .53
Discharged to new skilled nursing facility	26 (27)	13 (27)	13 (27)	RR^a^ 0.55; 95% CI, 0.27–1.12; *P* = .10

Abbreviations: B, fixed-effects estimates; DI, delirium index; HAC, hospital-acquired complications; HR, hazard ratio; IRR, incidence rate ratio; RR, relative risk.

a Adjusted for age, sex, frailty, cognition, accommodation, comorbidity and acute illness.

There were no differences in inpatient and 30-day mortality, or in new admission to a residential aged care facility.

### Adherence

The intervention was well tolerated, with >90% of doses administered in both groups (91.1% intranasal insulin vs 90.9% control). Overall, 85.6% of the participants were compliant (83.3% intranasal insulin vs 87.8% control).

### Safety

Adverse events (AEs) occurred in 42% of the participants (insulin: 41%, control: 43%), with 72 total events (insulin: 32, control: 40; see Appendix B in the online supplementary material). There were no statistically significant differences between groups in the proportion of participants experiencing an AE, or in the type and severity of AEs. Most were mild (CTCAE grades 1–2; insulin: 24, control: 36), with the most common being falls, aspiration pneumonia and urinary tract infections. Three grade 1 AEs were attributed to intranasal insulin: rhinorrhoea (*n* = 1), epistaxis (*n* = 1) and anosmia (*n* = 1).

There were 12 serious AEs (CTCAE grades 3–5; insulin: 8, control: 4). In the insulin group, serious AEs included hypernatremia (*n* = 2), aspiration pneumonia (*n* = 2), catheter-associated UTI (*n* = 1), lung infection (*n* = 1), hypokalaemia (*n* = 1) and hypophosphataemia (*n* = 1). Serious AEs in the control group were aspiration pneumonia (*n* = 3) and UTI (*n* = 1). All serious AEs were deemed unrelated or unlikely related to the intervention.

There were eight cases of level 1 hypoglycaemia (BSL 3.0–3.9 mmol/l, four per group; see Appendix C in the online supplementary material) but no glucose readings < 3.0 mmol/l [56]. Six participants experienced hyperglycaemia (insulin: 3, control: 3), with five having pre-existing type 2 diabetes (see Appendix D in the online supplementary material).

Twelve participants died during hospitalisation (see Appendix E in the online supplementary material); eight of these were attributed to delirium precipitants present on admission. No deaths were attributed to the study intervention, and no discernible patterns suggesting intervention-related risk were observed.

### Sensitivity analyses

As 11 patients died before delirium resolution. Delirium duration was analysed using death as a competing risk. Inpatient death did not significantly impact the primary outcome (HR 0.69; CI 0.43–1.12; *P* = .13).

In a per-protocol analysis including only compliant patients (*n* = 83; intranasal insulin: *n* = 40, control: *n* = 43), the median delirium duration was 4.61 days (IQR: 2.86–8.80) in the intranasal insulin group and 6.29 days (IQR: 2.96–9.76) in the control group (HR 0.61; CI 0.36–1.04; *P* = .07).

### Predefined exploratory analyses

Subgroup analysis based on the aetiology of delirium was performed on infectious precipitants only due insufficient power for other primary precipitants. Forty-nine participants had infection listed as the primary precipitant for delirium (intranasal insulin *n* = 23, control *n* = 26). The median delirium duration was 3.92 days (IQR: 2.87–9.22) in the intranasal insulin group and 7.32 days (IQR: 4.67–9.76) in the control group (HR 0.63; CI 0.31–1.30; *P* = .21).

## Discussion

This is the first study evaluating the use of intranasal insulin for the treatment of delirium in hospitalised older adults. Intranasal insulin was not associated with a statistically significant reduction in delirium duration. However, on prespecified subgroup analysis an age-related differential response was observed. Specifically, participants aged ≤88 years experienced a reduction in delirium duration with intranasal insulin compared to the control, while no differences were observed in participants aged >88 years. Participants treated with intranasal insulin had a shorter acute length of stay compared to the control. Adherence to the intervention was high and no safety concerns were identified. These findings underscore the potential of intranasal insulin as a targeted treatment for delirium in select patient populations and highlight the need for further investigation into the mechanisms driving age-related differences in treatment efficacy.

The rationale for this study was based on evidence of abnormal brain glucose metabolism during delirium. We found elevated cerebrospinal fluid (CSF) lactate and neuron-specific enolase in older medical patients with delirium, suggesting altered glucose metabolism [11]. Consistent with this, we demonstrated that delirium is associated with cerebral glucose hypometabolism on [^18^F]fluorodeoxyglucose positron emission tomography (FDG-PET) [12, 13]. Studies examining the CSF of older patients following hip fracture show elevated lactate and pyruvate during delirium, suggesting altered brain energy metabolism [14]. The same group found that delirium was associated with increased insulin resistance and ketones in patients with hip fracture [15]. Similarly, in another cohort of patients following hip fracture, delirium was associated with lower CSF insulin levels and greater insulin resistance [16].

Beyond its effects on cerebral glucose metabolism, intranasal insulin may target several key pathophysiological pathways implicated in delirium. In preclinical and human studies, intranasal insulin has been shown to modulate neuroinflammation by reducing proinflammatory cytokines such as TNF-α and IL-6 in the cortex, hippocampus, CSF and serum, while increasing the anti-inflammatory cytokine IL-10 and attenuating microglial overactivation—mechanisms relevant to interrupting the neuroinflammatory cascade implicated in delirium pathogenesis [19, 27, 30–32, 57, 58]. Intranasal insulin also regulates hypothalamic–pituitary–adrenal (HPA) axis activity by lowering stress-induced cortisol responses in human studies [29, 59]. Additionally, intranasal insulin enhances functional network connectivity, including increased resting-state connectivity between the hippocampus and the default mode network (DMN) and within the salience network [60–62]. Collectively, these findings highlight intranasal insulin’s potential to target multiple converging mechanisms implicated in delirium, particularly in a heterogeneous older population.

While no overall difference in delirium duration was noted in our study, the observed age-related response to intranasal insulin warrants further exploration. As brain insulin resistance increases with age [63], it is plausible that higher doses of insulin may be required in an older population. We used 20 IU of intranasal insulin; however, studies have demonstrated that higher doses can be given without inducing clinically significant hypoglycaemia [23]. A study on prevention of postoperative delirium found a dose-related response, with higher doses being associated with reduced incidence of delirium [28].

Our study also found no difference in delirium severity but did identify a significant decrease in acute length of stay favouring intranasal insulin. The drivers for this reduction warrant further interrogation. While a similar proportion of participants experienced a hospital-acquired complication, participants receiving placebo were more likely to experience multiple complications, though this was not statistically significant.

This study adds to a growing body of literature exploring the role of intranasal insulin in delirium. Single-centre studies have shown that intranasal insulin is effective in preventing postoperative delirium [27–32]. There are several reasons why our results may differ. First, we used long-acting insulin, based on earlier studies suggesting that it may confer some benefit [64]; more recent studies support the use of short-acting insulin [65]. Second, the type of nasal delivery device is critical. Craft *et al.*’s study on Alzheimer’s dementia demonstrated improved cognition with intranasal insulin using one nasal delivery device but not another [66]. Published prevention studies have used a different device to ours, potentially affecting the bioavailability. Third, demographic differences (e.g. younger participants, varied ethnicity and education) may have influenced the outcomes. Finally, our study included delirium of mixed aetiology, rather than postoperative delirium, which may involve distinct neural mechanisms.

### Strengths and limitations

The strengths of this study include its pragmatic and inclusive design; the trial population is representative of real-world patients. The participants were frail and at least 30% had a history of dementia—these patients are often excluded from clinical trials. Delirium assessments were conducted daily by trained research staff, 7 days a week, using input from electronic medical records and caregivers to enhance accuracy. While multiple daily assessments would have been ideal, resource constraints limited us to one per day. Retention was high during hospitalisation. Participants discharged with delirium were monitored at home for up to 1 week postdischarge to assess changes in condition following intervention cessation.

The limitations include the small sample size, which increases the likelihood of a type II error. Our initial power calculation did not account for the wide variability in the duration of delirium. The variability and outcome distributions observed in this study can guide more accurate power estimations in future larger studies.

Recognising the marked heterogeneity of delirium in older adults, we accounted for variability in baseline risk factors by adjusting for frailty, cognition, illness severity and comorbidity in our analyses. The small sample size precluded subgroup analyses by aetiology beyond infection, although most patients had multiple contributing factors for delirium. In addition, the number of participants in the nondementia and nonfrail subgroups was too small to support meaningful stratified analysis. The generalisability is limited by the study being single-site and only including patients with sufficient English language skills due to lack of timely access to formal interpreter services.

The device posed some limitations; it required training to use, cleaning procedures and intermittent troubleshooting. Consequently, the trial occurred on two hospital wards only, which hindered recruitment, particularly during the COVID-19 pandemic. Future studies should consider using more user-friendly devices [27], although head-to-head device studies have not been conducted and would provide critical insights. Recruitment was also halted and delayed due to staff leave and the COVID-19 pandemic.

We selected delirium duration as the primary outcome given its strong predictive value for adverse events in older adults, its intuitive clinical relevance and its endorsement by consensus guidelines for delirium trials in acute-care settings [67]. We report a sensitivity analysis with death as a competing risk. While composite outcomes such as ‘delirium-free days’ or ‘days alive and delirium-free’ offer the advantage of integrating mortality with recovery and have been used increasingly in ICU-based studies, these measures can conflate distinct negative outcomes (such as death and persistent delirium). This is particularly important in older populations, where both early mortality and persistent delirium are common and clinically divergent [5, 68]. Future trials should consider days alive and delirium-free as an outcome, while carefully delineating its interpretation. Using both approaches, supported by sensitivity analyses, may enhance the robustness and patient-centredness of intervention studies.

## Conclusion

Long-acting intranasal insulin did not reduce the duration of delirium in older people admitted to hospital with delirium, although it was safe and well tolerated. Patients treated with intranasal insulin, however, had a shorter length of stay compared to those treated with the control. The observed age-related response to intranasal insulin, where delirium duration was reduced in younger participants, warrants further investigation into the clinical potential of intranasal insulin in both the prevention and treatment of delirium.

## Supplementary Material

aa-25-1510-File002_afaf276

## Data Availability

Data available: yes. Data types: deidentified participant data. How to access data: data will be shared upon request by the corresponding author (a.nitchingham@unsw.edu.au). When available: following publication (TBA). Who can access the data: researchers whose proposed use of the data has been approved. Types of analyses: data will be made available for approved purposes. Mechanisms of data availability: with investigator support, after approval of a proposal and with a signed data access agreement.
