# Supplementary material for: Long-acting intranasal insulin for the treatment of delirium—a randomised clinical trial
Source: Age Ageing. 2025 Oct 14;54(10):afaf276. doi: 10.1093/ageing/afaf276 (PMC12526039; doi:10.1093/ageing/afaf276)
Supplement: aa-25-1510-File002_afaf276 [file aa-25-1510-file002_afaf276.docx]

**Long-Acting Intranasal Insulin for the Treatment of Delirium – A Randomised Clinical Trial**

**Supplementary Data**

**Appendix A: subgroup analysis by age ≤85 years**

**Appendix B: Adverse events, grouped using the Common Terminology Criteria for Adverse Events (CTCAE), Version 4.0 (2010)**

**Appendix C: Hypoglycaemic events**

**Appendix D: Hyperglycaemic events**

**Appendix E: Summary of inpatient mortality data**

**Appendix A**

Post-hoc subgroup analysis using age-cut off of ≤85 years^1^

**Age ≤85 years**

- *N* = 31 (Intranasal insulin: *n* = 13; Control: *n* = 18)
- Median duration of delirium (IQR):
   • Intranasal insulin: 4.58 days [3.02–6.89]
   • Control: 8.21 days [5.49–9.85]
- Hazard ratio (HR): 0.27 (95% CI 0.093–0.80)
- *P* = 0.017

**Age >85 years**

- *N* = 66 (Intranasal insulin: *n* = 35; Control: *n* = 31)
- Median duration of delirium (IQR):
   • Intranasal insulin: 5.19 days [2.87–11.11]
   • Control: 5.74 days [2.65–9.76]
- Hazard ratio (HR): 0.80 (95% CI 0.43–1.48)
- *P* = 0.45

^1^ Lau SWJ, Huang Y, Hsieh J, et al. Participation of Older Adults in Clinical Trials for New Drug Applications and Biologics License Applications From 2010 Through 2019. *JAMA Netw Open.* 2022;5(10):e2236149. doi:10.1001/jamanetworkopen.2022.36149

**Appendix B - Adverse events, grouped using the Common Terminology Criteria for Adverse Events (CTCAE), Version 4.0 (2010)**

| **CTCAE Group** | **Event** | **Intranasal Insulin (n=48)** | **Control (n=49)** |
| --- | --- | --- | --- |
| Blood and lymphatic disorders | Anaemia | 0 | 2 |
| Cardiac disorders | paroxysmal atrial tachycardia | 0 | 1 |
|  | heart failure | 0 | 2 |
| Gastrointestinal disorders | Gastrointestinal bleed | 1 | 0 |
| General disorders and administration site conditions | non-cardiac chest pain | 1 | 4 |
|  | left lower limb oedema | 0 | 1 |
| Infections and infestations | Urinary tract infection | 2 | 3 |
|  | Lung infection | 1 | 0 |
|  | Catheter related UTI | 1 | 0 |
| Injury, poisoning and procedural complications | Fall | 7 | 11 |
| Metabolism and nutrition disorders | hypocalcaemia | 1 | 0 |
|  | hypophosphatemia | 1 | 1 |
|  | hypernatremia | 4 | 0 |
|  | hyperglycaemia | 1 | 0 |
|  | hypokalaemia | 1 | 1 |
|  | hyperglycaemia | 3 | 3 |
| Nervous system disorders | extrapyramidal disorder | 0 | 1 |
|  | anosmia | 1 | 0 |
| Psychiatric disorders | delirium | 1 | 0 |
| Renal and urinary disorders | acute kidney injury | 1 | 3 |
| Respiratory, thoracic and mediastinal Disorder | aspiration pneumonia | 2 | 4 |
|  | epistaxis | 1 | 0 |
|  | lung infection | 1 | 0 |
| Skin and subcutaneous tissue disorders | pressure injury (other) | 1 | 2 |
| Vascular disorders | superficial thrombophlebitis | 0 | 1 |
|  | **Total:** | **32** | **40** |

Reference: U.S. Department of Health and Human Services, National Institutes of Health, National Cancer Institute. Common Terminology Criteria for Adverse Events (CTCAE) v4.0. Published May 28, 2009; Revised June 14, 2010. NIH Publication No. 09-5410.

**Appendix C: Hypoglycaemic events**

Hypoglycaemic events were graded using two criteria:

1. Common Terminology Criteria for Adverse Events (CTCAE), Version 4.0 (2010)^1^
   - for CTCAE hypoglycaemia gradings start at <3.0mmol/L
2. IHSG: International Hypoglycaemia Study Group^2^
   - Level 1: Glucose alert value of 3.9mmol/L or less
   - Level 2: Glucose alert value of <3.0mmol/L
   - Level 3: Severe hypoglyceamia indicates cognitive impairment requiring external assistance for recovery

| Intervention arm | Cause(s) of delirium | History of diabetes | Diabetic medications at time of hypoglycaemia | Compliant  (doses administered) | Time between last dose administered and hypoglycaemic event | BSL and management of hypoglycaemia | CTCAE grade | IHSG grade | Notes |
| --- | --- | --- | --- | --- | --- | --- | --- | --- | --- |
| Placebo | Sepsis  Falls  Malnutrition | No | Nil | Yes (18/19) | 1.5hours | **3.3mmol/L**  (9pm)  Oral glucose replacement | N/A | 1 | Sepsis / reduced oral intake |
| Placebo | Sepsis  Falls  Malnutrition | No | Nil | Yes (18/19) | 11hours | **3.7mmol/L**  (7am, fasting)  Oral + IV maintenance fluids switched to dextrose 5% 60mL per hour | N/A | 1 | Sepsis, reduced oral intake |
| Insulin | Urosepsis | Yes | Basal-bolus supplemental insulin sitagliptin. | Yes (2/2) | Nil given prior | **3.6mmol/L**  (7pm, pre-meal)  Oral glucose replacement | N/A | 1 | Reduced oral intake, on insulin, sepsis. |
| Insulin | Urosepsis  Fall | Yes | Nil  Gliclazide and metformin 3 days prior | Yes (10/10) | 12hours | **3.2mmol/L**  (8am, pre-meal)  Oral glucose replacement | N/A | 1 | Reduced oral intake, AKI previously given sulphonylurea |
| Placebo | Urinary tract infection | No | Nil | No (2/6) | Nil received prior | **3.8mmol/L**  (5pm, pre-meal)  Oral glucose replacement, maintenance fluids changes to 4% Dextrose and 1/5 normal saline | N/A | 1 | Reduced oral intake |
| Placebo | Urosepsis | Yes | Insulin aspart/insulin protamine 30/70 with breakfast, lunch, dinner | Yes (8/8) | 5 hours | **3.2mmol/L**  (1am)  Oral glucose replacement | N/A | 1 | Reduced oral intake on insulin |
| Insulin | Urosepsis  Urinary retention | No | Nil | No (14/19) | 13hours | **3.8mmol/L** (10am, reduced oral intake) | N/A | 1 | Reduced oral intake |
| Insulin | Urosepsis  Urinary retention | No | Nil | No (14/19) | 3hours | **3.7mmol/L** (1am)  Oral glucose replacement | N/A | 1 | Reduced oral intake |

**Appendix D: Hyperglycaemic events**

Hyperglycaemic events were graded using the CTCAE version 4, gradings are:

- Grade 1: Fasting glucose value >ULN - 160 mg/dL; Fasting glucose value >ULN - 8.9 mmol/L
- Grade 2: Fasting glucose value >160 - 250 mg/dL; Fasting glucose value >8.9 - 13.9 mmol/L
- Grade 3: >250 - 500 mg/dL; >13.9 - 27.8 mmol/L; hospitalization indicated
- Grade 4: >500 mg/dL; >27.8 mmol/L; life-threatening consequences
- Grade 5: Death

Many patients had multiple hyperglycaemic events during their hospital admission. The table indicates the highest recorded CTCAE grading.

| Intervention arm | Cause(s) of delirium | History of diabetes | Diabetic medications on admission | Compliant  (doses administered) | Temporal relationship to intervention | CTCAE grade |
| --- | --- | --- | --- | --- | --- | --- |
| Insulin | Urinary tract infection, fall | No | nil | Yes (28/29) | During intervention | 2 |
| Placebo | Fall with long lie, pain | Yes | Linagliptin  Metformin | Yes (7/7) | 4 days after intervention ceased | 2 |
| Placebo | Hyponatraemia, medications, urinary retention | Yes | Insulin  Metformin  Sitagliptin | Yes (10/11) | 24 hours after ceasing intervention | 2 |
| Placebo | Urinary tract infection, acute kidney injury | Yes | Metformin | Yes (19/20) | During intervention | 2 |
| Insulin | Urinary tract infection, constipation | Yes | Linagliptin  Metformin | Yes (8/10) | During and after intervention | 2 |
| Insulin | Guillain-Barre Syndrome | Yes | Insulin  Metformin  Gliclazide | Yes (8/8) | During and after intervention | 2 |

References:

^1^U.S. Department of Health and Human Services, National Institutes of Health, National Cancer Institute. Common Terminology Criteria for Adverse Events (CTCAE) v4.0. Published May 28, 2009; Revised June 14, 2010. NIH Publication No. 09-5410.

| **Appendix E: Summary of inpatient mortality data** | | | |
| --- | --- | --- | --- |
| **Cause of death^a^** | **Whole Cohort (n=97)** | **Intervention (n=48)** | **Control (n=49)** |
| pneumonia | 2 | 2 |  |
| aspiration pneumonia | 2 |  | 2 |
| Sepsis of unknown origin | 1 |  | 1 |
| ischaemic stroke | 2 | 2 |  |
| myocardial infarction | 1 |  | 1 |
| streptococcus bacteraemia | 1 | 1 |  |
| liver failure | 1 | 1 |  |
| disseminated intravascular coagulation | 1 |  | 1 |
| Guilliane-Barre Syndrome | 1 | 1 |  |
| **Total:** | **12** | **7** | **5** |

^a^Cause of death as recorded in the New South Wales Births, Deaths and Marriages Registration Act 1995 Medical Certificate of Cause of Death.
